# A dataset from a hydraulically actuated forestry crane for data-driven stress estimation (PATU655Stress)

**DOI:** 10.1016/j.dib.2026.112848

**Published:** 2026-05-20

**Authors:** Sohaib Mustafa Saeed, Victor Zhidchenko, Heikki Handroos

**Affiliations:** Laboratory of Intelligent Machines, LUT University, Yliopistonkatu 34, 53850 Lappeenranta, Finland

**Keywords:** Hydraulic machinery, Forestry crane, Strain gauges, Pressure sensing, Actuator kinematics, Structural health monitoring, PATU655

## Abstract

Hydraulic forestry cranes operate under continuously varying loads, where coupling between hydraulic actuation, mechanical motion, and structural response affects performance, safety, and fatigue life. Accurate estimation of internal states and structural stresses remains challenging due to nonlinear hydraulics, structural flexibility, and limited sensing, and publicly available experimental datasets from real machines are scarce. This paper presents PatuCrane655, an experimental dataset collected from a real PATU 655 forestry crane. The dataset includes time-synchronized measurements of joystick inputs, hydraulic pressures, actuator displacements, boom angles, and strain-gauge signals at structurally critical locations, recorded under multiple payloads, hydraulic settings, and crane configurations. To demonstrate its applicability, a baseline sequence-based LSTM model was applied to predict structural strains from measured inputs, showing that the dataset supports accurate data-driven stress estimation. The dataset is intended to support data-driven modelling, state estimation, and structural stress prediction for hydraulic cranes.

Specifications Table

**Table:** 

Subject	Engineering & Materials science
Specific subject area	Experimental dataset for hydraulic crane strain estimation using synchronized hydraulic, mechanical, and structural measurements for data-driven modelling.
Type of data	Raw (Time-series data) Tabular (.csv files) Figures (sensor layouts, crane geometry, sensor measurement plots)
Data collection	Data were collected from a PATU 655 forestry crane in the Laboratory of Intelligent Machines, LUT University. Sensors included strain gauges on lift and jib booms, pressure sensors in cylinder chambers, inclination sensors for boom angles, displacement sensors for cylinders, and joystick input signals. All signals were recorded using a single data acquisition system with a common timeline.
Data source location	Laboratory of Intelligent Machines, LUT University, Lappeenranta, Finland.
Data accessibility	Repository name: Mendeley Data Data identification number: 10.17632/rn5j42rpyr.1 Direct URL to data: https://data.mendeley.com/datasets/rn5j42rpyr/1
Related research article	None

## 1 Value of the data

• The dataset contains synchronized experimental measurements recorded from a real forestry crane (PATU655). The dataset contains variety of features such as operator inputs, hydraulic pressures, actuator displacements, boom angles, boom angular velocities, and strain-gauge signals from known critical points of crane structure. The PATU655 has been widely used in studies for modelling, state estimation, and digital twin applications [[1], [2], [3],^11^], but publicly available datasets that combine the hydraulic, mechanical and structural responses from this crane remain limited.
• The time-series structure of the data, for instance, covering valve commands, pressure dynamics, cylinder movements, and boom kinematics, enables its reuse for identification and dynamic analysis of hydraulic crane behaviour. These signals can be used to develop and validate multibody hydraulic models, including parameter estimation and observer-based approaches commonly applied to hydraulic machinery.
• The inclusion of stress measurements together with the operational signals and other significant features allow the data to be used for data driven stress estimation task. Multivariate learning models can be trained to map hydraulic and kinematic inputs to structural response and compared across different feature representations.
• The recordings span multiple payloads, supply pressure, voltage inputs, crane postures, and operating modes, which enables reuse for studies on model generalization under varying operating conditions.
• The dataset enables the development of the machine learning based surrogate modelling for stress prediction, which can be used as an alternative to computationally intensive finite element methods in real-time or continuous monitoring applications. Such models can be integrated into intelligent control frameworks to improve safety and extend useful life of the machines.

## 2 Background

The PATU665 Crane has been widely used as a reference system in studies on modelling, simulation, and digital twin development of hydraulically actuated mobile machinery. Prior work has reported detailed multibody-hydraulic formulations, including actuator pressure flow dynamics, geometric parameters, and inertial properties [1,5,6]. Studies conducted on PATU655 Crane have also shown that multibody dynamics combined with state estimation methods, such as Unscented Kalman Filter, can reduce the number of required sensors by reconstructing unmeasured system states from limited measurements [1]. However, the lack of publicly available experimental datasets limits independent validation, comparison of estimation strategies, and the development of hybrid data-driven and physics-based approaches.

Machinery condition monitoring increasingly relies on measured sensor data to identify abnormal behaviour and assess structural or operational conditions. Data driven systems have shown valid proof of concept utilizing experimental data for industrial applications [19]. In general, it is found that measured operational and structural signals can also serve as a basis for future degradation- or digital twin-driven monitoring studies [20]. In parallel, machine learning methods for structural response estimation have been explored in other engineering domains, such as aerospace and civil and has significance for different applications and therefore offer potential for similar applications in heavy machinery [15].

The present dataset is introduced to support reproducibility, benchmarking, and the development of data-driven and hybrid modelling for hydraulic heavy crane systems.

## 3 Data Description

The dataset was recorded using a PATU 655 hydraulically actuated forestry crane installed in the Laboratory of Intelligent Machines, at LUT University, Lappeenranta, Finland. The PATU 655 crane has served as a benchmark platform in a wide range of previous studies focusing on modelling, simulation, control, digital twin development, and lifecycle analysis of mobile hydraulic machinery. Early simulation and fatigue-related investigations on this crane were reported by Mikkola [7], where the simulation model was used for identification of fatigue parameters and lifetime assessment. Subsequent studies developed detailed multibody and hydraulic models of the crane for control and dynamic analysis, including modelling and control of flexible hydraulic manipulators [8,9].

More recent works have established the PATU 655 crane as a reference system for digital twin and real-time simulation studies. Faster-than-real-time simulation frameworks and digital twin implementations were presented in [2,10], demonstrating real-time capable dynamic models of the hydraulically actuated crane. These concepts were further extended to lifecycle management and IoT-enabled digital twins in [11] and combined with physics-based digital twin formulations in [3]. The crane has also been used in studies on boom trajectory optimization and energy efficiency under electrification [4], as well as in investigations of advanced electrohydraulic actuation concepts, including valve-free operation of the lift cylinder [12]. In addition, the crane has been used in lifecycle and sustainability analyses, such as the life cycle assessment of lifetime extension strategies reported in [14]. Together, these studies provide a well-established experimental and modelling background that motivates the use of the PATU 655 crane as a reference system for the present dataset. The dataset is designed so it can be used for validation against multibody/FEM/digital twin models. It is not intended as dedicated fatigue life dataset tied to single assessment framework or fatigue parameters, but rather operational measured and strain values that can support future fatigue related analysis utilizing appropriate damage assessment methods.

The crane consists of a pillar, a lift boom, and a jib boom with an extension boom fixed inside it. The dominant planar motion is generated by two double-acting hydraulic cylinders: the lift cylinder actuating the lift boom and the jib cylinder actuating the jib boom. The mechanical layout, kinematic structure, and actuator configuration correspond to the industrial crane design described in manufacturer specifications [16] and in previous modelling studies based on Mevea and Simulink implementations of the same crane [13]. These references provide the geometric dimensions, joint definitions, and mass properties that form the basis for consistent simulation studies.

**Fig. 1:**
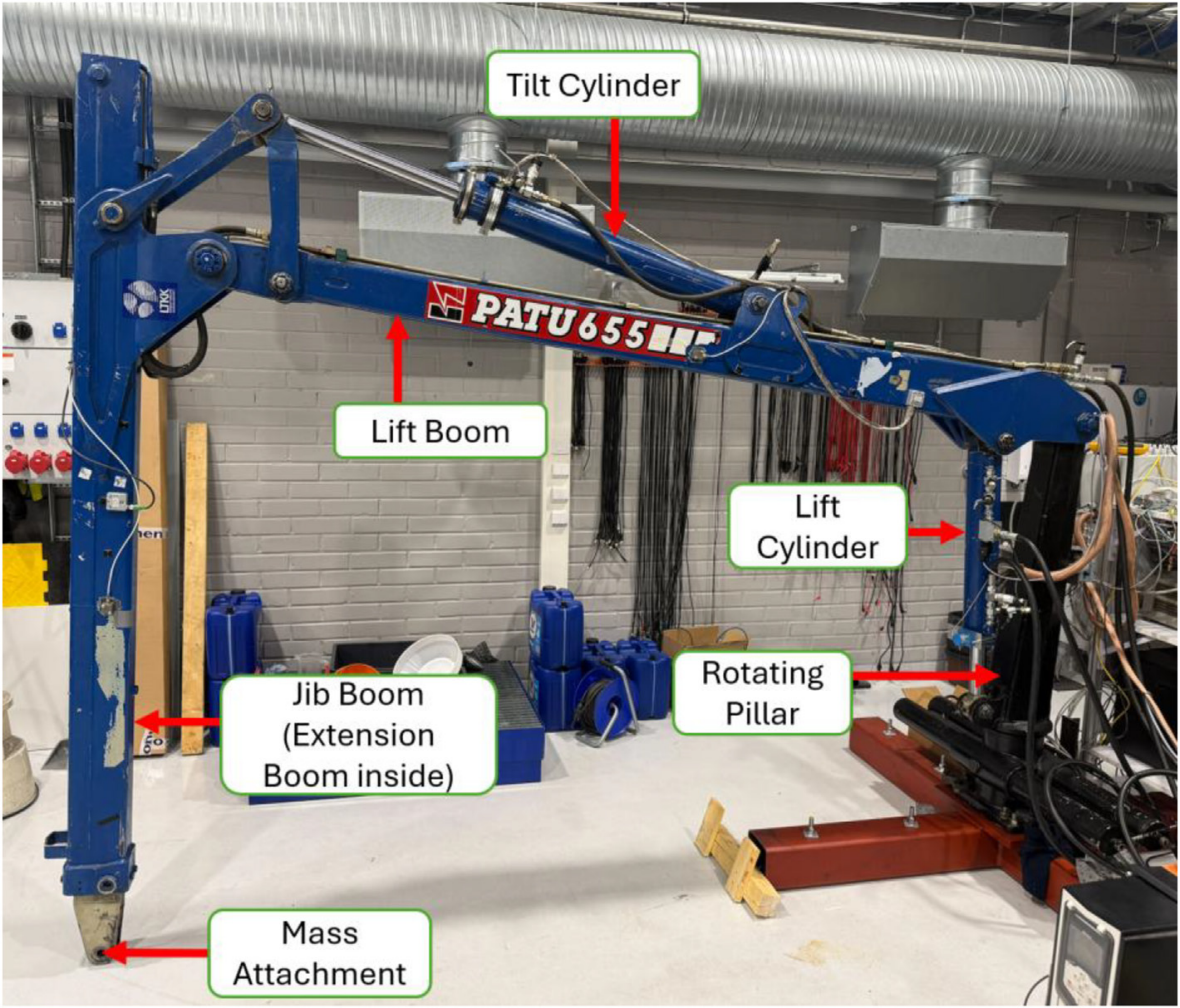
Experimental crane setup used for measurements.

The crane geometry, joint configuration, and actuator arrangement follow the modelling conventions reported in [1,5,6]. The geometric dimensions, mass properties, and hydraulics of the crane links correspond to those reported in [1], enabling consistency between the dataset and previously published simulation and state-estimation models. The hydraulic circuit dynamics and the coupling between multibody dynamics and hydraulic actuators are aligned with the computationally efficient formulations presented in [5] and the fluid power circuit modelling methods described in [6]. The general multibody system formulations with electromechanical and hydraulic actuation are consistent with the approaches reported in [17]. These modelling frameworks ensure that the recorded measurements can be directly used for validation of physics-based, reduced-order, and hybrid modelling approaches. Fig. 1 shows the experimental crane setup used for data collection. The main structural components and hydraulic actuators are annotated. Locations of the strain gauges mounted on the lift and jib booms are detailed separately in Fig. 3. The experimental configuration follows earlier laboratory implementations of the PATU 655 crane for state estimation and digital twin validation [1,2,3,11], which enables direct comparison between the present dataset and previously published simulation, estimation, and lifecycle management studies.

The hydraulic actuation system follows a conventional valve-controlled double-acting cylinder configuration. Each actuator is supplied through a proportional directional control valve connected to a common pressure supply and tank return line. The pressure–flow dynamics, valve behaviour, and actuator force relations used to interpret the recorded signals correspond to the formulations described in [1,5,6]. Cylinder dimensions and hydraulic parameters used for derived force computation match those reported in [1].

### 3.1 Recorded features

All measurements were collected through a centralized data acquisition driven by a common timing reference, enabling consistent time alignment between hydraulic, mechanical and structural channels.

The operator control inputs consist of voltages applied to the proportional valves that supply pressure to lift and jib actuators. Actuator kinematics are represented by the measured displacements of the lift and jib hydraulic cylinders. The hydraulic states include piston-side and rod-side pressures for both cylinders, as well as the supply and return line pressures of the hydraulic system. Mechanical states are described by the lift and jib boom angles together with their corresponding angular velocities. Structural response is captured through strain measurements obtained from five strain gauges mounted on the crane structure. The data are intended to capture the interaction between valve commands, pressure in cylinder chambers, actuator motion, boom configuration, and resulting structural loading. Table 1 summarizes the recorded variables, their physical meaning, units, and their availability across the two operating modes.

**Table 1:** Recorded variables and operating modes in the PATU655Stress dataset.

Variable group	Parameter (column name)	Physical meaning	Units
Operator control inputs	Lift valve command (Ulift[V])	Joystick / proportional valve command voltage for the lift cylinder directional valve	V
	Jib valve command (Ujib[V]))	Joystick / proportional valve command voltage for the jib cylinder directional valve	V
Actuator kinematics	Lift cylinder displacement (S1[mm])	Lift cylinder piston stroke / position ()	mm
	Jib cylinder displacement (S2[mm])	Jib (Jibjib) cylinder piston stroke / position ()	mm
Hydraulic states	Lift piston-side pressure (PA1[bar])	Piston-side (chamber A) pressure of lift cylinder,	bar
	Lift rod-side pressure (, PB1[bar])	Rod-side (chamber B) pressure of lift cylinder,	bar
	Jib piston-side pressure (PA2[bar])	Piston-side (chamber A) pressure of jib (Jib) cylinder,	bar
	Jib rod-side pressure (PB2[bar])	Rod-side (chamber B) pressure of jib (Jib) cylinder,	bar
	Supply pressure (PS[bar])	Pump/supply line pressure of the hydraulic circuit	bar
	Tank pressure (PT[bar])	Tank/return line pressure of the hydraulic circuit	bar
Mechanical states	Lift boom angle (Incl1[deg])	Lift boom inclination angle	degrees
	Jib boom angle (Incl2[deg])	Jib boom inclination angle	degrees
	Lift boom angular velocity (LiftGyrZ[degps])	Angular velocity of lift boom	Degrees per second
	Jib boom angular velocity (JibGyrZ[degps])	Angular velocity of jib boom	Degrees per second
Structural response	Strain channels (Strain1LTL–Strain5LTRFar) [ppm]	Strain-gauge measurements mapped to	Micro strains

The crane was operated under two main motion patterns:

(i) Continuous motion, where the crane executes repeated lifting and lowering trajectories.
(ii) Step-type motion, where discrete command steps are applied to excite transient hydraulic and mechanical dynamics.

**Fig. 2:**
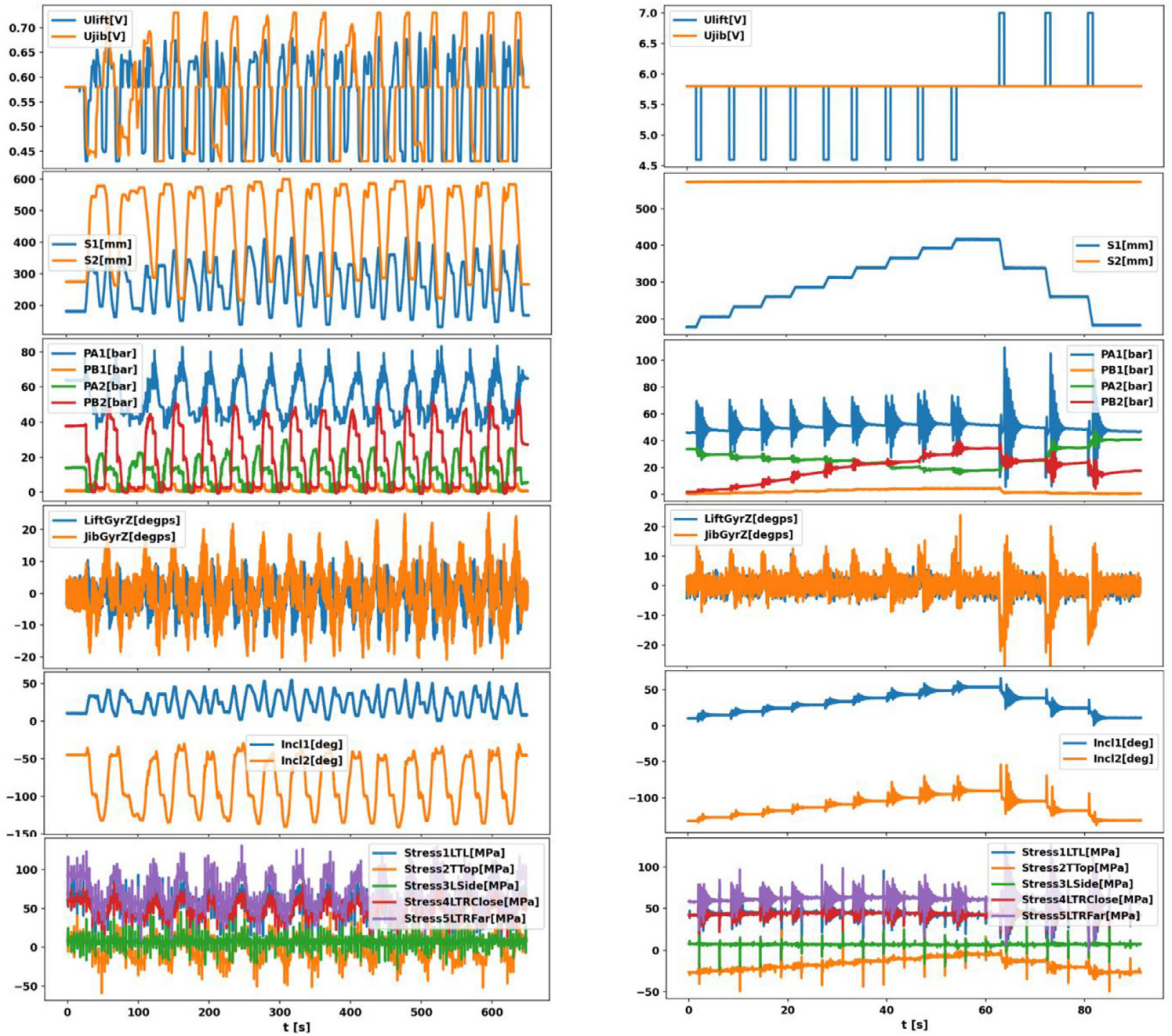
Representative time-series signals recorded from the PATU 655 crane under continuous operation (left) and step-type movements (right), including input signals, actuator displacements, cylinder pressures, angular velocities, boom angles, and stress values.

Fig. 2 presents representative examples of the recorded signals under two operating modes. The left column corresponds to continuous crane operation, where the crane is driven through repetitive motion sequences. The right column shows step-type crane movements, where discrete command changes are applied. In both cases, the plots illustrate the synchronized evolution of control inputs, hydraulic pressures, actuator motion, boom kinematics, and strain responses.

### 3.2 Strain measurement channels and sensor placement

To capture the structural response of the crane under hydraulic actuation and payload loading, five strain gauges were mounted at structurally relevant locations on the lift and jib (Jib) booms. The sensor placement was selected based on crane geometry, expected structural loading and scope of present experiments, with objective of capturing both global bending-dominated behaviour and localized high-response regions near the main joints of the lift and jib booms, which are dominant load-bearing members during the considered planar crane motion, while rotational motion of the pillar was not included in the experimental campaign. In this context, multibody simulation-based approaches can also support sensor placement by identifying locations that are most sensitive to the dominant mechanical behaviour and loading conditions of structures [18].

**Fig. 3:**
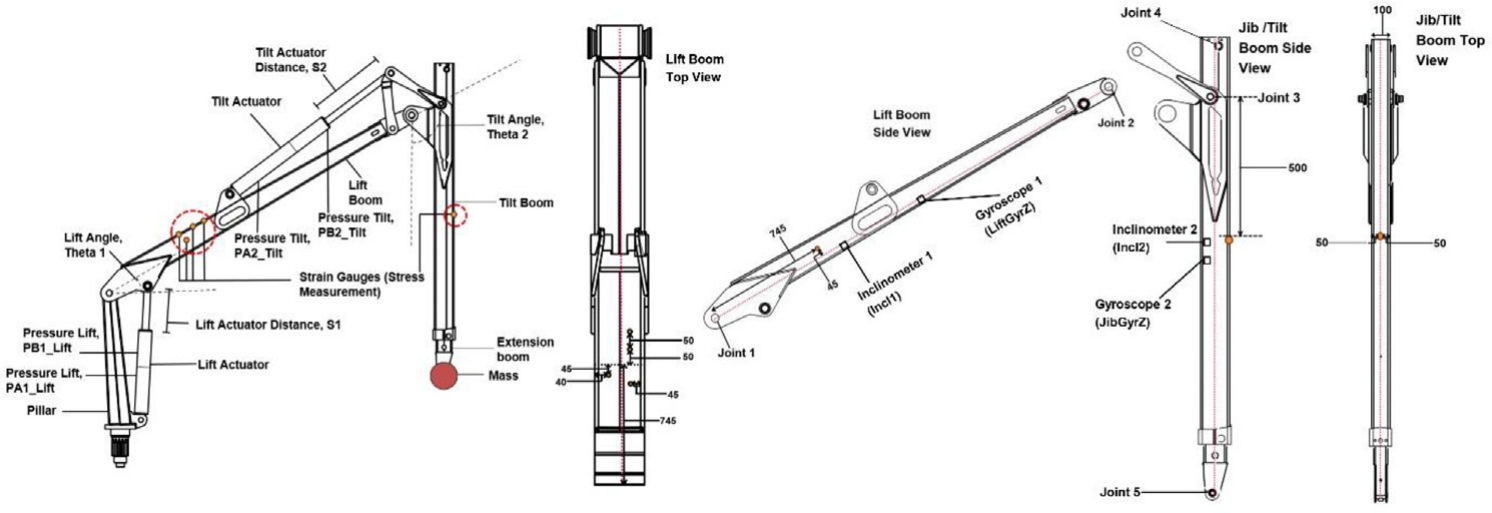
Geometry and strain gauge locations of the PATU 655 crane used in PATU655Stress.

Fig. 3 illustrates the crane geometry and sensor instrumentation used in the PATU655Stress dataset, showing the overall geometry and sensor locations on the lift and jib booms, hydraulic cylinders, and joints. Detailed top and side views show the exact positions of the strain gauges relative to the main articulation joints. Four strain gauges were distributed on the lift boom to capture bending-related strain response and asymmetry around the neutral axis. One strain gauge was placed on the jib boom near the articulation joint, where significant stress variations occur during crane motion and payload handling. The strain gauges directly measure local surface strain at the selected locations under real operating conditions. This is consistent with established experimental stress analysis practice, where strain is measured and stress is subsequently derived using material laws [22]. However, for this present study, young modulus as $210\times 10^{9}$, and standard conversion of stress/strain can be applied using Hooke’s law within the elastic region.

### 3.3 Experimental coverage and operating conditions

A total of 108 recordings were collected under varying crane configurations, payload masses, and hydraulic operating conditions in order to capture a broad and practically relevant range of crane dynamics and structural loading scenarios. Mechanical configurations and hydraulic actuation settings were varied across the experiments, while sensor instrumentation and acquisition procedures were kept constant. The crane was operated over multiple combinations of boom angles, hydraulic supply pressures, and flow-rate settings, and with different payload masses attached at the crane tip. The variation in these parameters enables analysis of crane behavior under different loading levels and working postures, as well as evaluation of model robustness across operating conditions. The experimental groups are summarized in Table 2, which lists the boom angle ranges, hydraulic settings, payload masses, and the number of recordings per group. All experiments were conducted under a typical indoor laboratory condition approximately room temperature (about 20–23 °C).

**Table 2:** Summary of experimental recordings in the PATU655Stress dataset.

ID	Angle ranges	Hydraulic settings	Payload	Recordings
Exp-1	θ₁ ∈ {0°–17°}, θ₂ ∈ {30°–115°}	PS: 80–130 bar; QS: 80/100 L/min	95 kg	29
Exp-2	same as Exp-1	same as Exp-1	180 kg	44
Exp-3	same as Exp-1	same as Exp-1	278 kg	33
Exp-4*	same as Exp-1	same as Exp-1	148 kg	2

* Unseen test recordings.

The first three experimental groups correspond to the primary payload masses used for model development and training, while the fourth group contains recordings with an intermediate payload mass that was not used during training of baseline LSTM model and is intended for evaluation and generalization testing. Since the main contribution of the paper is the dataset, users may define alternative test-train splits depending on the intended application, for example by payload, motion type, operating conditions etc. The number of recordings differ across experimental group because dataset reflects the actual experimental campaign rather than a deliberately balanced benchmark design. The 148KG payload has least number of recordings as it was intended for unseen test scenario but includes both type of operational movements and enough temporal-sequential data to acquire context.

### 3.4 Raw data format and organization

**Fig. 4:**
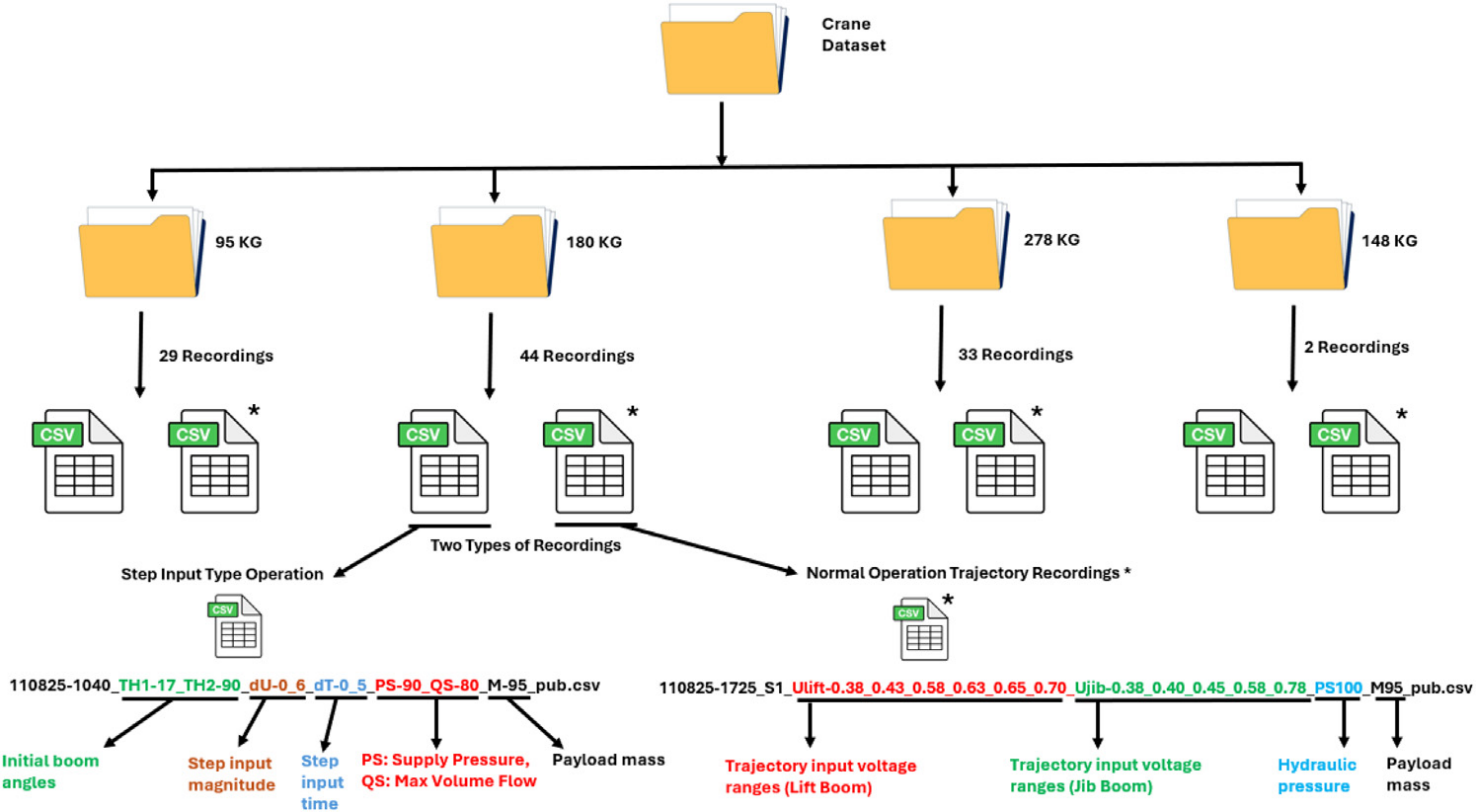
Folder structures and data description.

The dataset is organized in a hierarchical directory structure based on payload mass, as illustrated in Fig. 4. The main dataset directory contains four subfolders corresponding to the payload masses used in the experiments: 95 kg, 148 kg, 180 kg, and 278 kg. Each payload folder contains multiple comma-separated value (CSV) files, where each file represents an individual experimental recording. These CSV files contain recorded measurements of operator inputs, hydraulic pressures, actuator displacements, boom angles, angular velocities, and strain-gauge signals.

For two types of recordings: step input recordings and trajectory recordings, the step input recordings include discrete voltage steps that are applied to the hydraulic actuators to excite transient system dynamics. The corresponding file names encode key experimental parameters, including TH1 and TH2 representing the initial lift and jib boom inclination angles and ranges, dU denoting the step input magnitude, dT indicating the step input time, PS and QS representing hydraulic supply and return pressures, and M indicating the payload mass. In trajectory recordings, the crane is operated using continuous actuator input trajectories. In these files, the parameters Ulift and Ujib represent the actuator input voltage ranges applied to the lift and jib cylinders, respectively, while PS denotes the hydraulic supply pressure and M indicates the payload mass. To distinguish trajectory recordings from step input recordings in Fig. 4, trajectory recording files are marked with an asterisk (*) symbol in the figure. This symbol is used only for visual clarification in the figure and is not part of the actual file names or folder structure in the dataset.

## 4 Experimental Design, Materials and Methods

The experimental platform enabled controlled actuation of the crane and synchronized recording of hydraulic, mechanical, and structural response signals. The setup supported repeatable execution of predefined test sequences while keeping instrumentation and acquisition settings consistent across all recordings.

### 4.1 Sensor instrumentation and measured variables

All measurements except the control signals were made with analogue transducers that converted physical quantities to electrical signals (voltage or current). The measurement signals were collected by a data acquisition system, which converted them into digital representation and transmitted them to the control PC for display to the user and saving to disk. The validation of sensor recording is based on the physical calibration of sensors by use of real instrumentation crane.

### 4.2 Data acquisition system

**Fig. 5:**
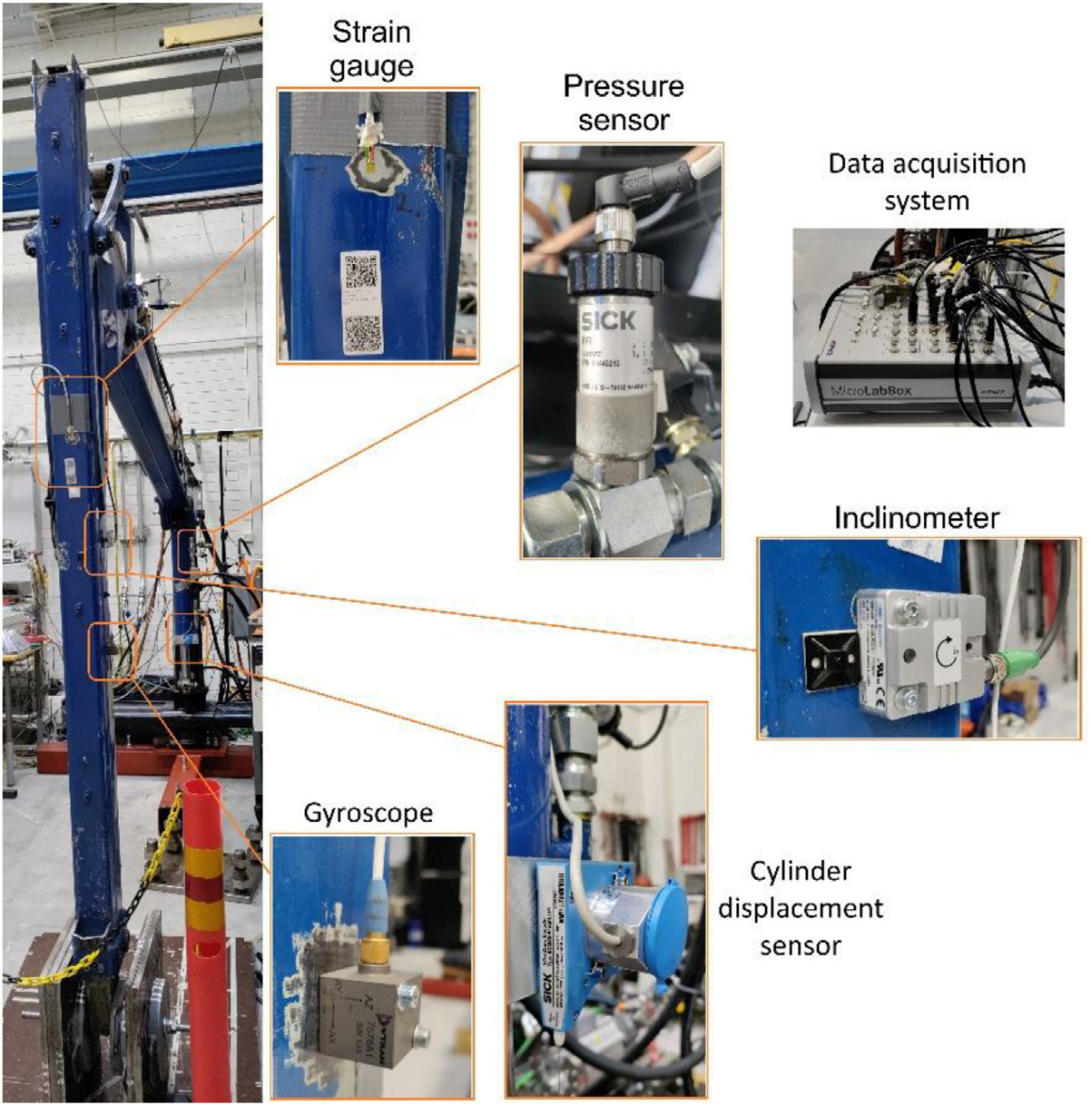
Sensors and the data acquisition system used in experiments.

We used the dSPACE MicroLabBox data acquisition system to interact with all the sensors and actuators (Fig. 5). It converted the sensor analog signals received on its input ports into digital form with a 16-bit analog-to-digital converter. It also converted the digital control signals received from the Python control script into analog signals on its output ports. The data acquisition system communicated with the Python control script through a direct point-to-point Ethernet connection using the UDP protocol. The communication with the control script occurred every 17 ms to correspond to the refresh rate of the user interface. The control script received the measurement data, combined them with the input control signals provided by the user, added a timestamp, and saved all the data to a file. The resulting sampling rate for the data saved to the files was 58.8 samples per second.

### 4.3 Control inputs

The voltage signals for controlling the hydraulic valves were generated differently in three modes of operation. In continuous-motion and step-type experiments, the operator was setting specific values for the lift and jib valve signals using a keyboard and a graphical user interface. The Python control script implementing the user interface transmitted these values to the data acquisition system and saved them to a file. In joystick control mode, another Python script interfacing with joysticks received the signals from the joysticks in digital form and transmitted the received values to the control script, which sent them to the data acquisition system and saved them to a file.

### 4.4 Pressure measurement

The pressure sensors measured the pressure referenced to the ambient atmospheric pressure. They were installed in each chamber of the lift and jib cylinder. In addition, the pressure sensors were installed on the supply and tank lines. We used SICK PFT-SRB250AG1SSAAMSSZ sensors that converted the value of the pressure in the range 0 bar … 250 bar to the output current in the range 4 mA … 20 mA (Fig. 5). A current-to-voltage converter converted the current to voltage signal in the range 0 V … 10 V that was connected to the input port of the data acquisition system.

### 4.5 Cylinder displacement measurement

To measure cylinder displacement, we used SICK wire draw encoders BCG05-K1KM01PP installed at the lift and jib cylinders (Fig. 5). The sensor consists of a wire draw mechanism and an encoder. The rotation of the drum, which is proportional to the length being measured, is recorded and output by an encoder as a current signal in the range 4 mA … 20 mA. This signal was converted to the 0 V … 10 V voltage by a current-to-voltage converter to connect it to the data acquisition system. The sensor was calibrated to provide zero output when the cylinder was fully contracted. To obtain the actual distance between the cylinder joints, the value of the corresponding cylinder displacement in the dataset should be added to the contracted cylinder length (0.81 m for the lift cylinder and 1.05 m for the tilt cylinder). These offsets are fixed do not vary across experiments, allowing users to compute total cylinder length directly when needed.

### 4.6 Measurement of the boom angles

Angles between each boom and the horizon were measured by the inclinometers Baumer GIM500R-M136.FC4.A (Fig. 5). The sensors were installed on the sides of the booms. The sensor at the lift boom was aligned with a line passing through the joints Lift-boom-Pillar and Lift-boom-Tilt-boom (joints 1 and 2 at Fig. 3, correspondingly). The sensor measured zero degrees when this line was parallel to the horizon. The sensor at the jib boom was aligned with a line passing through the joints Extension-cylinder-Jib-boom and Extension-boom-Payload (joints 4 and 5 at Fig. 3). Both sensors converted the measured inclination angle in the range 0 degrees … 360 degrees to the output current 4 mA … 20 mA. The output signal was connected to the data acquisition system through the current-to-voltage converter similar to the sensors described above.

### 4.7 Angular velocity measurement

Angular velocity of the booms was measured by the gyroscopes Dytran 7576A1 (Fig. 5). These sensors convert angular velocity in the range −50 deg/s … 50 deg/s to the voltage signal with the sensitivity 25 mV per degree per second (±10%). The voltage signal was directly connected to the data acquisition system, that is why the angular velocity data in the dataset are quite noisy for the range of real velocities of the booms. The gyroscopes were installed in the middle of the line segments connecting the Joint 1 and Joint 2 at the lift boom and Joint 4 and Joint 4 at the jib boom (Fig. 3). Positive angular velocity corresponds to counterclockwise rotation. No digital filtering was applied to gyroscopic signals before inclusion to the published dataset; the angular velocity channels are provided as recorded by the acquisition system. Preprocessing filtering may be applied by users depending on the application.

### 4.8 Strain measurement

Strain gauges installed in five locations measured an extensional strain on the surface of the lift and jib booms. We used HBM 1-LY41–3/120 strain gauges connected to the HBM measurement amplifier KWS3073. The amplifier output provided voltage signal to the data acquisition system with the sensitivity 1 V per 300 microstrains (parts per million or ppm). The locations of the strain gauges are presented in Fig. 3. The strain values provided in the dataset represent direct experimental measurements obtained from the installed strain gauges and were used as the target structural-response signals in the example machine learning study.

### 4.9 Experimental scenario and test conditions

The experiments were organized to systematically excite the coupled hydraulic actuation, mechanical motion, and structural deformation of the crane. Test sequences were designed to produce diverse temporal response patterns, including gradual load transitions and rapid dynamic changes. This approach allows investigation of system behaviour across different dynamic regimes and supports development and validation of predictive and estiation models. Independent recordings were included to enable objective evaluation of model generalization under conditions not used during model development.

### 4.10 Data cleaning and machine learning feasibility

To demonstrate the suitability of the dataset for data driven applications, the raw strain measurements were pre-processed as part of example machine learning workflow. The published dataset itself contains the original raw recordings.

### 4.11 Strain signal cleaning

Raw strain signals contained impulsive peaks caused by electromagnetic interference, acquisition artifacts, and occasional backlash-related impacts of the fixed internally extension boom. To reduce influence of these non/physical disturbances in the machine learning example, a multi-criterion outlier detection approach was applied to the strain channels:

1. Temporal gradient: Detects abrupt changes between consecutive samples
2. Rolling median deviation: Identifies samples deviating strongly from the local trend
3. Modified z-score: Provides robust outlier detection

**Fig. 6:**
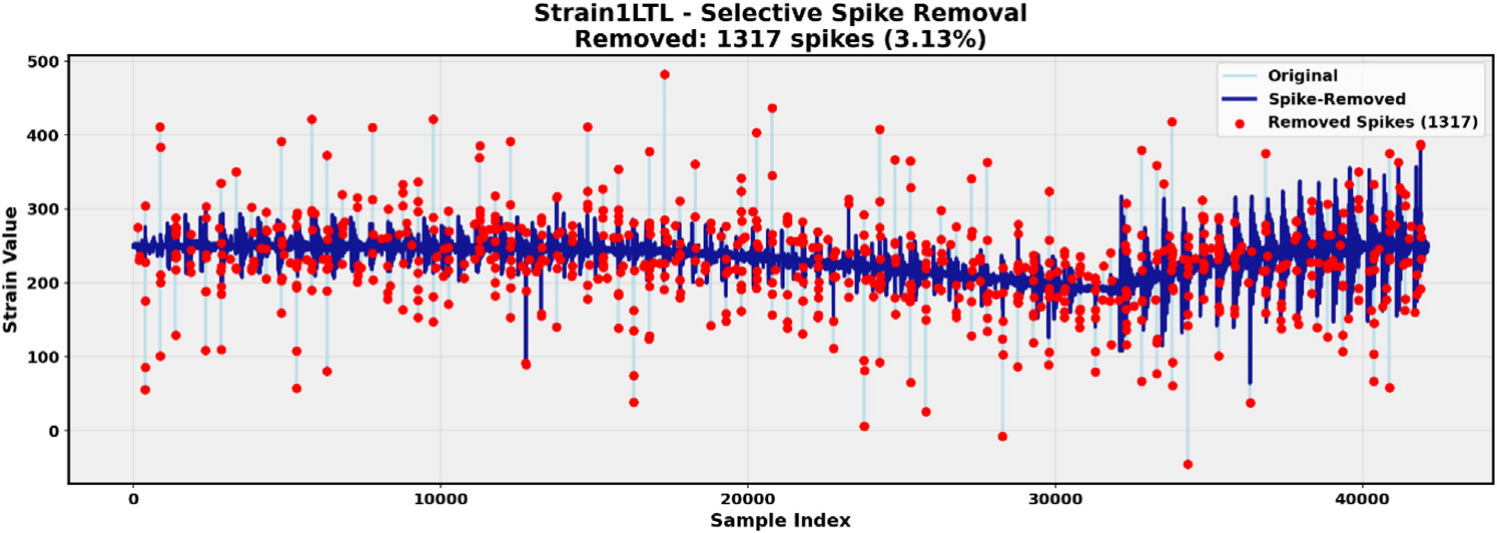
Example of strain preprocessing showing raw signal with impulsive peaks, detected outliers (highlighted in red), and cleaned signal after interpolation-based replacement.

Samples flagged by at least two criteria were classified as outliers and replaced via linear interpolation between neighboring valid samples. The preprocessing parameters, including the rolling median window size, were selected empirically to supress clearly non-physical impulsive peaks while preserving the overall strain response used while no sensitivity analysis was formally performed in this case. As, the data contains the raw recordings, users can utilize the alternative preprocessing techniques as fit for their applications. This preprocessing step was used only for the machine learning feasibility demonstration and was not applied to alter the published raw dataset. This approach preserves the underlying stress dynamics while removing non-physical artifacts. Fig. 6 illustrates the cleaning process, showing the raw strain signal with impulsive peaks, detected outlier samples, and the resulting cleaned signal.

### 4.12 Machine learning validation

**Fig. 7:**
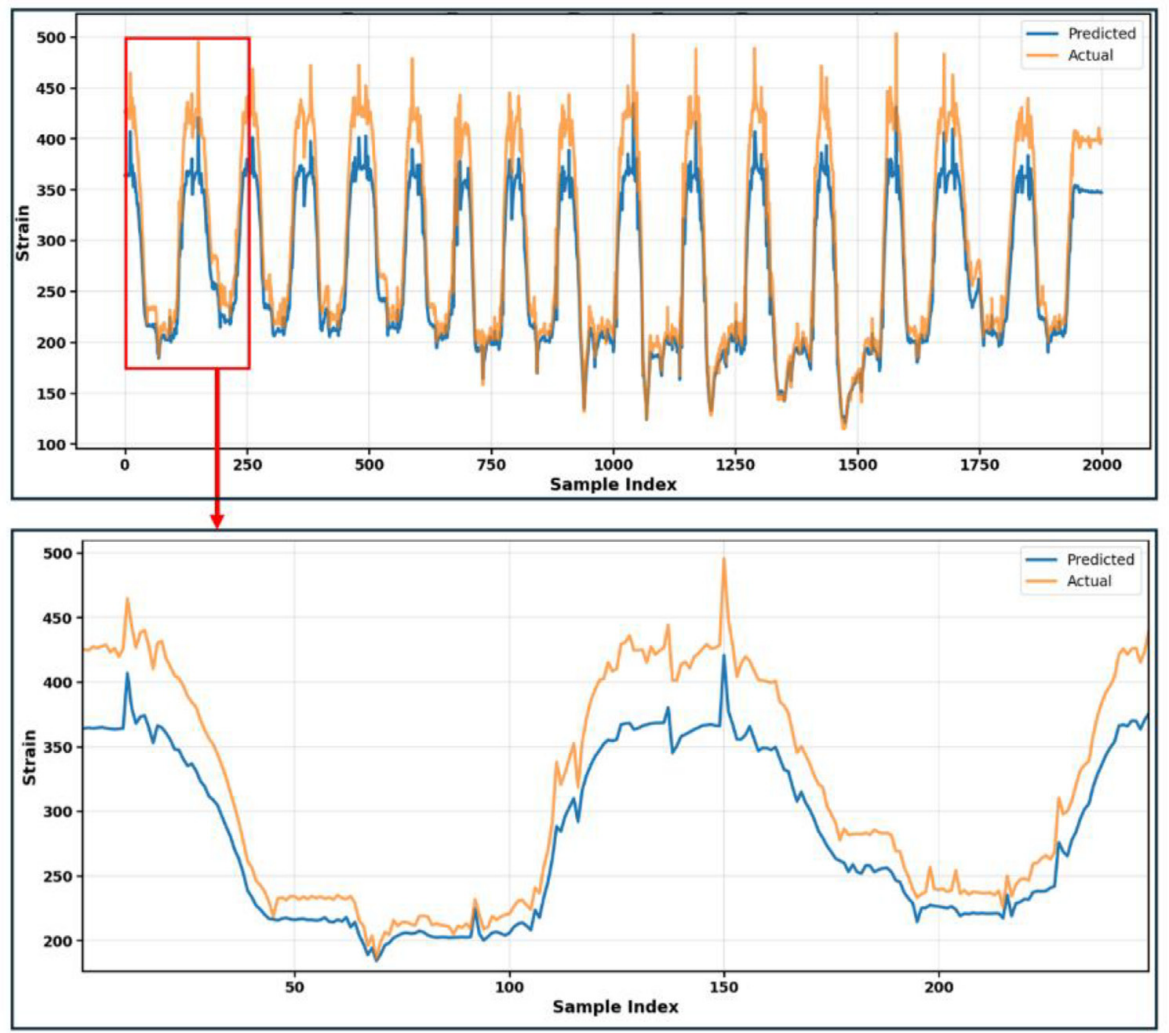
Predicted and measured strain responses for continuous motion with unseen 148 kg payload. Upper panel shows the full-time sequence; lower panel provides a zoomed view of the highlighted region.

The overall workflow consisted of four stages: data collection, data preparation, application-oriented preprocessing, and machine learning model training/testing. In the data collection stage, synchronized measurements were recorded and stored in raw format. The data preparation stage included basic inspection of the recorded signals and removal of clearly non-physical disturbances for the example modelling workflow. The application-oriented preprocessing stage included feature engineering, where hydraulic cylinder pressures were converted into estimated actuator forces, since these forces are directly related to boom loading and structural response [21]. To validate the dataset's utility for predictive modelling, a baseline Long Short-Term Memory (LSTM) network as widely used for its ability to capture temporal dependencies in sequential data was trained to predict structural strain responses from operational measurements (actuator forces, boom angles, valve commands, and payload mass). The proposed model uses a stacked LSTM-based neural network to capture temporal relationships between operational inputs and structural strain. The input sequence, consisting of actuator forces, boom angles, valve commands, and payload mass, is processed through three LSTM layers with 128, 64, and 32 units, respectively, with dropout applied after each layer to reduce overfitting. The final LSTM output is passed to a fully connected dense layer with ReLU activation for feature extraction, followed by a regression layer that predicts strain values for five channels simultaneously. The model was tested on recorded unseen operating conditions with a 148 kg payload, because the unseen payload group (EXP-4) contains only two recordings, reported results are interpreted as illustrative feasibility check rather than a statistically significant assessment of model generalization. Table 3 presents the prediction performance across the five strain channels, and Fig. 7 shows the comparison between predicted and measured strain responses. The strain channel names (Strain1LTL–Strain5LTRFar) correspond to individual strain gauges installed on the crane structure, where Strain1LTL to Strain4LTR are located on the lift boom and Strain5LTRFar is located on the jib boom, as defined in Table 1 and spatially illustrated in Fig. 3.

**Table 3:** Baseline LSTM performance on unseen 148 kg payload (average across two unseen recordings EXP-4).

Strain Channels	MSE (ppm²)	MAE(ppm)	RMSE (ppm)	R²
Strain1LTL	604.32	19.95	24.06	0.58
Strain2TTop	189.67	9.16	13.53	0.88
Strain3LSide	79.48	2.97	8.90	0.07
Strain4LTR	301.28	13.89	16.79	0.63
Strain5LTRFar	863.23	23.02	28.05	0.64

The variation in MSE, MAE, RMSE, and $R^{2}$ values across the strain channels is influenced by differences in signal characteristics, including amplitude ranges, variability and noise levels. The prediction performance also varied across the strain channels the lower $R^{2}$ was observed for Strain3LSide, Strain4LTR and Strain5LTR5Far because of the sensor placement and signal characteristics. Strain3LSide, mounted on the side of the lift boom, showed smaller variation because crane motion was mainly planner and bending dominated, while also being more affected by noise peaks. On the other hand, Strain5LTRFar and Strain4LTR, showed lower prediction because of dominant structural loading during normal operation was concentrated more on the known critical locations.

These preliminary results demonstrate that the PatuCrane655 dataset contains sufficient information for learning-based structural response prediction and while strain does not directly represent structural damage, it is a fundamental quantity used in fatigue and structural analysis and can be used to derive stress cycles and assess structural loading when combined with appropriate material and modelling assumptions. The varying performance across channels indicates opportunities for advanced modelling approaches, which will be explored in future work. The dataset can also support the development, and validation of control-oriented, physics-based, and hybrid modelling approaches using real crane measurements. Since many boom-type hydraulic cranes share a similar articulated structure consisting of a base, lift boom, and jib boom actuated by hydraulic cylinders, the dataset may also support the development of control and trajectory optimization methods, as well as physics-informed learning approaches, for similar hydraulic crane architectures.

## Limitations

The experiments were conducted in a controlled laboratory environment with stable temperature and humidity. The dataset does not reflect outdoor environmental variations (temperature changes, wind, rain) that may affect hydraulic performance and structural response during field operation.

Only two recordings were available for unseen-payload evaluation (148 kg). Although these sequences contain sufficient dynamic content, additional independent test recordings would enable high level statistical model generalization assessment. Training data include only three discrete payload levels (95 kg, 180 kg, 278 kg), which may limit interpolation to intermediate masses and extrapolation to extreme loads. Although the internal extension boom was fixed, there was a backlash resulted in small movements and ocasional impacts between the extension and jib boom, which were not measured but produced small spikes in the strain data. The dataset mainly represents planar dynamics of the lift and jib booms and does not capture the full kinematic and structural complexity of the complete crane. In particular, slewing motion and associated out-of-plane loading were not included in this experimental setup. Only two operating modes (continuous and step-type motion) were considered, with strain gauges at limited structural locations. Additional operating modes, diverse trajectories, rotational movement, and measurement from pillar, base structure, or slewing system would further improve coverage od dynamic behaviour and localized structured response.

## Ethics Statement

The authors have read and comply with the ethical requirements for publication in Data in Brief. The present work does not involve human participants, animal experiments, or any data collected from social media platforms.

## CRediT Author Statement

**Sohaib Mustafa Saeed:** Conceptualization, Methodology, Software, Formal analysis, Investigation, Data curation, Writing – Original Draft, Visualization. **Victor Zhidchenko:** Conceptualization, Methodology, Investigation, Resources, Supervision, Writing – Review & Editing, Project administration. **Heikki Handroos:** Conceptualization, Supervision, Resources, Funding acquisition, Writing – Review & Editing.

## Data Availability

Mendeley DataA Dataset from a Hydraulically Actuated Forestry Crane for Data-Driven Stress Estimation (PATU655Stress) (Original data)
